# Irradiated Riboflavin Diminishes the Aggressiveness of Melanoma *In Vitro* and *In Vivo*

**DOI:** 10.1371/journal.pone.0054269

**Published:** 2013-01-16

**Authors:** Daisy Machado, Silvia M. Shishido, Karla C. S. Queiroz, Diogo N. Oliveira, Ana L. C. Faria, Rodrigo R. Catharino, C. Arnold Spek, Carmen V. Ferreira

**Affiliations:** 1 Laboratory of Bioassays and Signal Transduction, Department of Biochemistry, Institute of Biology, University of Campinas, Campinas, São Paulo, Brazil; 2 Center for Experimental and Molecular Medicine, Academic Medical Center, University of Amsterdam, Meibergdreef, Amsterdam, The Netherlands; 3 Laboratory Innovare of Biomarkers, Faculty of Medical Sciences, University of Campinas, Campinas, São Paulo, Brazil; IDI, Istituto Dermopatico dell'Immacolata, Italy

## Abstract

Melanoma is one of the most aggressive skin cancers due to its high capacity to metastasize. Treatment of metastatic melanomas is challenging for clinicians, as most therapeutic agents have failed to demonstrate improved survival. Thus, new candidates with antimetastatic activity are much needed. Riboavin (RF) is a component of the vitamin B complex and a potent photosensitizer. Previously, our group showed that the RF photoproducts (iRF) have potential as an antitumoral agent. Hence, we investigated the capacity of iRF on modulating melanoma B16F10 cells aggressiveness *in vitro* and *in vivo*. iRF decreases B16F10 cells survival by inhibiting mTOR as well as Src kinase. Moreover, melanoma cell migration was disrupted after treatment with iRF, mainly by inhibition of metalloproteinase (MMP) activity and expression, and by increasing TIMP expression. Interestingly, we observed that the Hedgehog (HH) pathway was inhibited by iRF. Two mediators of HH signaling, GLI1 and PTCH, were downregulated, while SUFU expression (an inhibitor of this cascade) was enhanced. Furthermore, inhibition of HH pathway signaling by cyclopamine and Gant 61 potentiated the antiproliferative action of RF. Accordingly, when a HH ligand was applied, the effect of iRF was almost completely abrogated. Our findings indicate that Hedgehog pathway is involved on the modulation of melanoma cell aggressiveness by iRF. Moreover, iRF treatment decreased pulmonary tumor formation in a murine experimental metastasis model. Research to clarify the molecular action of flavins, *in vivo*, is currently in progress. Taken together, the present data provides evidence that riboflavin photoproducts may provide potential candidates for improving the efficiency of melanoma treatment.

## Introduction

Melanoma is the most aggressive form of skin cancer and causes around 75% of all deaths related to skin cancer [1]. Melanoma incidence rates have been increasing for at least 30 years, and for 2012 it is estimated that there are nearly 1 million melanoma survivors living in the United States, and an additional 76,250 individuals will be diagnosed [2]. Usually melanoma displays a rapid systemic dissemination, high capacity of metastasis, and it is extremely refractory to conventional antineoplastic treatments [3], [4], [5]. Patients with melanoma metastasis have a poor prognosis, for instance, when the metastatic site is in the lung, patients have a median survival of 12 months [3].

Metastasis is responsible for as much as 90% of cancer-associated mortality. The complex metastatic cascade can be conceptually organized and simplified into two major phases: (i) physical translocation of a cancer cell from the primary tumor to the microenvironment of a distant tissue, followed by (ii) colonization, involving complex processes that are still unclear [6]. Degradation and remodeling of extracellular matrix (EMC) and basement membrane by proteolytic enzymes facilitates angiogenesis, tumor cell invasion, and are essential steps in the process of metastasis [7], [8]. The basement membrane is mainly composed of type IV collagen and fibronectin [9], [10], which can be degraded by matrix metalloproteinases (MMPs). MMPs are a family of zinc-dependent matrix-degrading enzymes, and include gelatinases, collagenases, stromelysins, as well as others [10]. In this study, we focused on gelatinases (MMP-2 and MMP-9) that degradate a number of ECM molecules such as type IV, V and XI collagens, laminin and aggrecan core protein [10]. Moreover, MMPs also process and activate signaling molecules, including growth factors and cytokines, making these factors more accessible to target cells by either liberating them from the ECM or by shedding them from the cell surface [11]. Some tumor cells such as present in pancreas, bladder, colorectal, ovarian and prostate cancer secrete detectable amounts of MMP-2 and MMP-9, making them potential cancer biomarkers [11]. Under physiological conditions, MMP activity is inhibited specifically and reversibly by a group of structurally related endogenous inhibitors known as tissue inhibitors of metalloproteases (TIMPs) [11].

Besides metastasis, the high capacity of proliferation and survival are important factors for melanoma aggressiveness. Protein kinase AKT controls several cellular functions ranging from cell survival, proliferation, differentiation, and cell motility to tumor suppression and apoptosis [12], [13], [14], [15]. The major mediator of AKT activation is PI3K, which leads to inhibition of pro-apoptotic signaling molecules or induction of anti-apoptotic molecules enabling a growth advantage to the tumor cells [14]. AKT is overexpressed in nearly 60% of advanced-stage melanomas in patients [16]. One of the major targets of AKT is the mammalian target of rapamycin (mTOR) which is also a key regulator of cell growth, proliferation, differentiation, survival [17], tumor cell motility, invasion and cancer metastasis [18]. The multiprotein complex mTORC1 contains mTOR, RAPTOR (regulatory associated protein of mTOR), mLST8 (also known as GβL), and PRAS40 (proline-rich AKT substrate 40 kDa) [19]. S6K1 is extensively described as a mTORC1substrate and when activated by mTORC1, it promotes protein synthesis by phosphorylating PDCD4 (targeting it for degradation) [19]. Despite being an indirect measure of mTORC1, S6 phosphorylation is widely used in research and in the clinic as a biomarker of mTORC1 activity [19]. Another pivotal kinase for tumor aggressiveness is Src, a nonreceptor protein tyrosine kinase, which is considered as a key player in tumor progression providing oncogenic signals for cell survival, epithelial-mesenchymal transition, mitogenesis, invasion, angiogenesis, and metastasis [20]. Many of the mentioned downstream effects of Src are mediated through recruitment and activation of focal adhesion kinase and mitogen activated protein kinase (ERK). Additionally, Src is one of the main regulators of the melanoma invasion driven by proteases [21].

Biological effects of hedgehog (HH) are mediated through a pathway that involves binding to patched (PTCH), thereby releasing Smoothened (SMO) from inhibition; resulting in the activation of GLI transcription factors, which mediate the induction of HH target genes. HH signaling has been related to events promoting cancer progression in different tumor types, including melanoma, breast, ovarian, skin, prostate, and pancreatic cancers [22], [23], [24], [25], [26]. Two human PTCH receptors are known: PTCH-1 and PTCH-2. Although both receptors have been implicated in a variety of diseases, most cancers in which HH signaling is activated have been shown to occur through PTCH-1 [22], [25]. In addition, GLI1 transcriptionally upregulates chemoattractants and pro-angiogenic proteins, such as fibroblast growth factor, vascular endothelial growth factor (VEGF), and angiopoietin, that act both proximal and distal thereby promoting angiogenesis, cell migration and/or invasion [27], [28]. Interestingly, Das and colleagues [29] reported that HH-GLI1 signaling transcriptionally upregulates osteopontin, indicating a potential role of HH pathway in melanoma invasion. Currently, the HH pathway is associated with the development of resistance to conventional chemotherapy in solid tumors as well as in certain types of leukemias. HH and GLI1 are overexpressed in the majority of residual solid tumor after chemoradiotherapy, suggesting that HH signaling contributes to chemotherapy resistance in such tumors [30].

Riboflavin (RF), 7,8-dimethyl-10-ribityl-isoalloxazine, is a component of the B2 vitamin complex present in aerobic organisms as the precursor of the redox coenzymes flavin mononucleotide and flavin adenine dinucleotide. Light sensitivity adds to the broad biochemical importance of RF. iRF consists of compounds with diverse biochemical properties and has many pharmaceutical implications, such as reducing pathogens and inactivating white blood cells in donated blood products [31]. Given these applications, RF is one of the most widely studied compounds since its discovery [32].

The major photoproducts of RF are 7,8-dimethyl-10-(formylmethyl)isoalloxazine (formylmethylflavin), lumichrome, 2′-ketoriboflavin 4′-ketoriboflavin and, lumiflavin [33], [34]. It has been shown by Ohkawa *et al* (1983) [35] and Chastain and McCormick (1987) [36] that RF photodecomposition can occur *in situ* in the human body. Our group showed that irradiated RF (iRF) has a strong anti-tumoral effect on leukemia cells [37], as well as anti-proliferative and anti-metastatic effects on solid tumors [34]. We described that the iRF anti-tumoral molecular mechanism, is based on the modulation of a set of proteins that culminates in lower cancer cells aggressiveness and induction of apoptosis [34], [37].

In this study, we demonstrate that iRF modulates many aspects involved in the metastatic features of melanoma such as invasion, migration and proliferation. We propose that iRF limits melanoma aggressiveness by negative modulation of Src kinase, mTor and HH pathways, as well as metaloproteinases. Importantly, reinforcing our hypothesis of the iRF anti-tumoral potential, these flavins were also able to inhibit pulmonary tumor formation in a murine experimental metastasis model.

## Materials and Methods

### Material

MTT and Riboflavin (≥ 98%) were purchased from Sigma. Antibodies were purchased from Cell Signaling (mTOR/2983, p-mTOR/2971, p-p70 S6K/9206S, p-Src/2101 and cIAP/4952), Abcam (GLI1/ab92611) and Santa Cruz (SUFU/sc-10933, p70 S6K/sc-230, MMP-9/sc-6841, MMP-2/sc-6838, TIMP-1/sc-6832, bcl-2/sc-7382, actin/sc-1616-R, PTCH-1/sc-6149 and c-Src N-16/sc-19). Cyclopamine and Gant 61 were obtained from LC Laboratories and Calbiochem, respectively. The cell proliferation BrdU kit was obtained from Roche Applied Science.

### Riboflavin Irradiation

A solution of 150 µM riboflavin in DMEM medium was placed in a Petri dish and irradiated with UVA light (UVL - 28 EL; 365 nm UV; 8 W/115 V∼80 Hz/0.32 A) at a dose of 9 J/cm^2^.

### Identification of Photoproducts of Riboflavin in Culture Medium

Sample Preparation. 150 µM riboflavin and iRF samples (100.0 µL) were diluted in a flask with a 1∶1 solution of H_2_O:MeOH, 0.1% formic acid (J. T. Baker, Pennsylvania, USA) to a final volume of 1.0 mL.

ESI-MS Monitoring. Samples were directly infused at a flow rate of 10.0 µL min^-1^. ESI-MS and ESI-MS/MS in the positive ion mode were acquired using a LTQ-Orbitrap Discovery (Thermo Scientific, Bremen, Germany) instrument with 30000 mass resolution in the Orbitrap mass analyzer. Typical operating conditions were 10 (arb) sheath gas, 3.5 kV spray voltage, 43 V capillary voltage, 275°C capillary temperature and 112 V tube lens. High-resolution was the most important identification parameter [38], and the mass accuracy was calculated based on ppm shifts. Ion-selection and collisions were performed both by the ion-trap, followed by mass analysis of product-ions by the high-resolution Orbitrap analyzer and ESI-MS/MS were collected by causing collision-induced dissociation (CID) of the mass-selected protonated molecules using helium (He) as the buffer gas and collision energies from 10 to 25 eV. ESI-MS were acquired over a *m/z* range of 200 to 500.

### Cell Culture

B16F10 (mouse melanoma) and 2H11 (mouse umbilical vein endothelial cells) cell lines were obtained from American Type Culture Collection (ATCC; Manassas, VA), HaCaT (human keratinocyte) cells were kindly provided by Dr. Liudmila L. Kodach (Academic Medical Center, Amsterdam University). All cell lines were routinely grown in DMEM supplemented with 10% fetal bovine serum (FBS) and antibiotics (100 U/mL penicillin, 10 µg/mL streptomycin) in a humidified incubator with 5% carbon dioxide, at 37°C.

### Pre-treatment of B16F10 with HH Pathway Inhibitors

B16F10 cells were plated in 96 well plates and 24 h later the cells were pre-treated with cyclopamine (5 µM), Gant 61 (5 µM) or SHH (0.5 µM) for 6 h. Thereafter, iRF was added and cells were incubated for 24 h. Cell viability was analyzed by a MTT reduction assay as described below.

### MTT Reduction Assay

The medium was removed from the cells and 100 µL of thiazol blue tetrazolium bromide (MTT) solution (0.5 mg/mL in FBS free culture medium) was added to each well. After incubation for 2 h at 37°C, the MTT solution was removed and the formazan crystals were solubilized in 100 µL of DMSO. The plate was shaken for 5 min on a plate shaker and the absorbance was measured at λ = 570 nm in a microplate reader (Synergy HT, BioTek) [39]. The measured absorbance at λ = 570 nm was normalized to % of control. This value was calculated by multiplying the absorbance of a treated well by 100 and dividing it by the average absorbance of a control well, which was considered 100%.

### BrdU Incorporation Assay

B16F10 cells were grown to 70% confluence in a black 96 wells microplate with flat and clear bottom after which they were treated with different concentrations of irradiation RF for 24 h. Next, BrdU incorporation was quantified according to the BrdU kit manufacturer’s instructions (Roche). In brief, the cells were incubated with BrdU for 3 h. After the B16F10 cells were fixated and incubated with anti-BrdU for 90 minutes. Next, the assay substrate was added and incubated for 5 minutes and chemiluminescence was measured in a microplate reader (Synergy HT, BioTek). The values of measured chemiluminescence were normalized to % of control. These values were calculated multiplying the chemiluminescence of treated wells by 100 and dividing them by the average chemiluminescence of control wells, which were considered 100%.

### Colony Assay

Cells (1×10^2^) were seeded in flat-bottom 6 well plates and treated with different concentrations of iRF. After treatment for 2 or 10 days the cells were incubated with 0.2% crystal violet and 2.5% glyceraldehyde for 30 min. The number of colonies were counted using a stereomicroscope.

### Scratch Assays

B16F10 cells were seeded in a six well plate. After 24 h a scratch was made in the confluent monolayers using a 200 µL tip. The scratched cells were treated with different concentrations of iRF for 16 hours and images of six different fields per scratched area were made using a phase-contrast microscope. For each condition three independent experiments were performed. The size of the remaining scratch was plotted as a ratio of the scratch area at t = 0 h compared to t = 16 h.

### Transwell Migration Assay

Cells were labelled for 1 h with 10 µmol/L CellTracker Green 5-chloromethylfluorescein diacetate (CMFDA) (Molecular Probes, Eugene, OR, USA) in serum-free medium. The dye was fixed by 1 h incubation in medium with 10% FCS. Subsequently, cells were resuspended in serum free medium with or without iRF and transferred to 8-µm pore size HTS FluoroBlok cell culture inserts (BD Falcon, Franklin Lakes, NJ). Medium containing 20% FBS was added to the bottom well and cell migration was assessed as described previously [40], [41]. In brief, fluorescence values representing the number of cells on the bottom side of the insert were measured during 120 cycles (each cycle comprising four readings spanning 2 min) at 37°C. The raw fluorescence data was corrected for background fluorescence and fluorophore bleaching.

### Transmigration Assay

Endothelial 2H11 cells were plated in 24 well plate Fluoroblock™ inserts and allowed to grow until100% confluent. Next, labeled B16F10 cells (10^6^ cell/mL) were added and transmigration was assessed using the same procedure as described above for the transwell migration assays, except that fluorescence was measured at 3 and 18 h.

### Western Blotting Analysis

After treatment with iRF, the medium was removed and the cells were washed with cold physiological solution. Cells were lysed in cell lysis buffer: 50 mM Tris [tris(hydroxymethyl)aminomethane]-HCl, pH 7.4, 1% Tween 20, 0.25% sodium deoxycholate, 150 mM NaCl, 1 mM EGTA (ethylene glycol tetraacetic acid), 1 mM O-vanadate, 1 mM NaF and protease inhibitors (1 µg/mL aprotinin, 10 µg/mL leupeptin and 1 mM 4-(2-amino-ethyl)-benzolsulfonyl-uoride-hydrochloride) for 2 h on ice. Protein extracts were cleared by centrifugation, and the protein concentration was determined using the BCATM Protein Assay Kit (Thermo Scientific). An equal volume of 2× sodium dodecyl sulfide (SDS) gel loading buffer: 100 mM Tris-HCl (pH 6.8), 200 mM dithiothreitol (DTT), 4% SDS, 0.1% bromophenol blue and 20% glycerol was added to the samples and boiled for 10 min. Cell extracts, corresponding to 3×10^5^ cells, were resolved by SDS–polyacrylamide gel (12%) electrophoresis (PAGE) and transferred to polyvinylidene diuoride (PVDF) membranes. Membranes were blocked in bovine serum albumin (1%) in Tris-buffered saline (TBS)-Tween 20 (0.05%) and incubated overnight at 4°C with the appropriate primary antibody at 1∶1000 dilution. Next, the membranes were washed in TBS-Tween 20 (0.05%), incubated with 1∶1000 of the appropriate HRP-conjugated secondary antibody (DakoCytomation) for 1 h, and blots were imaged using LumiLight Plus ECL (Roche, Basel, Switzerland) on a LAS-4000 imaging system.

### Zymographic Analysis

Proteolytic activity of MMP-2 and MMP-9 was assayed by gelatin zymography as described by Souza *et al* [42]. In brief, culture medium was collected and stored at −20°C until further use. Samples were diluted in non-reducing buffer (0.1 M Tris-HCl, pH 6.8, 20% glycerol, 1% SDS and 0.001% bromophenol blue), and the volume of the samples loaded was proportional to the protein concentration. The samples were resolved by SDS–polyacrylamide gel (10%) and 4% gelatin (Sigma-Aldrich). Protein renaturation was done using 2% Triton X-100 for 1 h followed by incubation with 50 mM Tris-HCl and 10 mM CaCl2 (pH 7.4) at 37°C for 18 h. Gels were stained with 0.5% Coomassie blue G 250 for 30 min and then washed in a 30% methanol and 10% glacial acetic acid solution.

### Animals

Eight to ten week-old C57Bl/6 male mice (Charles River, Maastricht, The Netherlands) were maintained at the animal care facility of the Academic Medical Centre, Amsterdam, The Netherlands according to institutional guidelines. Animal procedures were carried out in compliance with Institutional Standards for Human Care and Use of Laboratory Animals. The institutional Animal Care and Use Committee approved all experiments (protocol number DIX102294-1).

### Experimental Pulmonary Metastasis Model

Cancer cells (suspended in 200 µl PBS) were injected into the lateral tail vein as described previously [43], [44], [45]. In the first experiment, 3×10^5^ cancer cells were administered per mouse. Afterwards, the animals were treated (i.p.) 3 times per week with PBS, riboflavin (0.1, 0.2 mg/kg) or iRF (0.1, 0.2 mg/kg). After 14 days, mice were sacrificed and lungs were prepared as described before [46]. Secondary tumor formation on the surface of the lungs was counted macroscopically in a blinded fashion with respect to the intervention. Experiments were performed with 8 mice per group.

### Statistical Evaluation

Statistical analysis was performed using GraphPad Prism version 4.03. All experiments were conducted in triplicate and the results were expressed as the means ± standard deviation (SD). Data from each assay were analyzed statistically by ANOVA. Multiple comparisons among groups were determined with the Tukey test. Differences were considered significant when the *p* value was less than 0.05.

## Results

### Photoproducts of Riboflavin in Culture Medium

From the UVA irradiated DMEM (Dulbecco's Modified Eagle Medium) dissolved RF, riboflavin and the following photoproducts of riboflavin were identified: Lumichrome 7,8-Dimethylisoalloxazine, Formylmethylflavin and Carboxymethylflavin (Table 1).

**Table 1:** iRF composition measurements obtained by high-resolution mass spectrometry.

Metabolite	TheoreticalMass	ExperimentalMass	Mass Error(ppm)	RelativeIntensity	RelativeConcentration
Riboflavin	399.1275	399.1275	0	8739.464	11.2191
Lumiflavine (LF)	279.0852	NF	NF	NF	NF
7,8-Dimethylisoalloxazine	267.0852	267.0851	–0.37441236	266.6761	0.3423
Carboxymethylflavin (CMF)	323.0751	323.0747	–1.238102225	21.55509	0.0277
Lumichrome (LC)	265.0696	265.0696	0	67427.78	86.5589
Formylmethylflavin (FMF)	307.0802	307.0800	–0.651295655	106.9098	0.1372

### iRF Reduces Murine Melanoma Cells but not Human Keratinocytes Cell Viability

**Figure 1:**
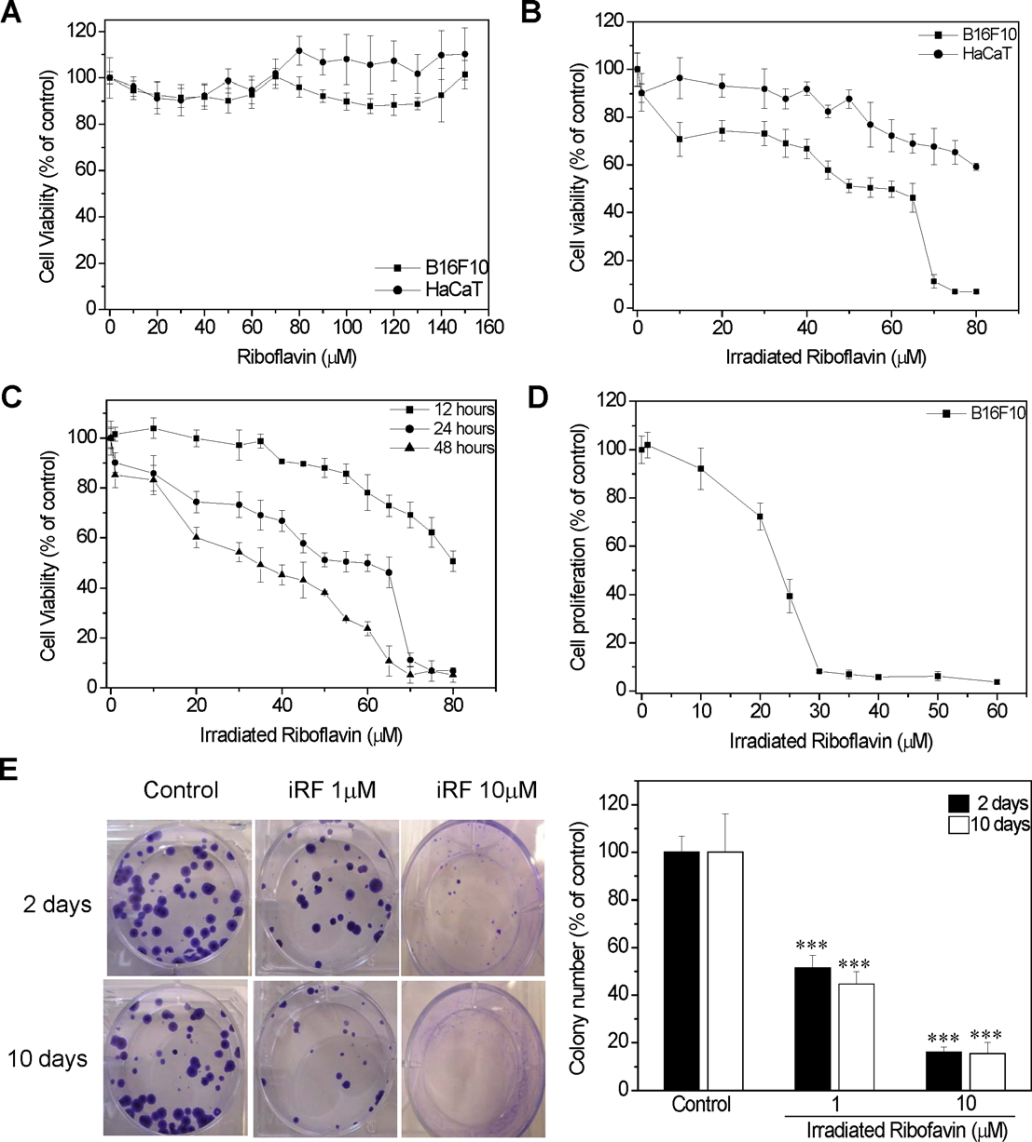
Effects of iRF on B16F10 cell viability. (A) B16F10 and HaCaT cells were treated with different concentrations of RF and (B) iRF for 24 h, and cell viability was assessed by MTT reduction assay. (C) B16F10 were treated with iRF for 12, 24 and 48 h. (D) Cell proliferation evaluation by BrdU assay of B16F10 treated with iRF and (E) representative pictures of colony assay, and the number of colonies. B16F10 cells were treated for 2 and 10 days. The extent of MTT reduction and colony assay content only medium with SFB was considered as 100%. The results represent the means ± SD (n = 9). p<0.01, p<0.001 versus control.

From the MTT reduction assay it was observed that non-irradiated RF did not affect the viability of murine melanoma cells (B16F10) nor human keratinocytes (HaCaT) - (Figure 1A), while melanoma cells were sensitive for iRF (IC_50_ = 50 µM), human keratinocyte cells (HaCaT) were much less sensitive, cell viability dropped only 35% in the concentration of 80 µM (Figure 1B). In addition, melanoma cell viability was also evaluated after 12 and 48 h of treatment which resulted in IC50 values of 80 and 35 µM, respectively (Fig. 1C).

Next, the iRF effect on melanoma proliferation rate was determined by BrdU and colony assays. Figure 1D shows that 20 µM of iRF was able reduce BrdU incorporation by 50%. The colony assay is based on the principle that certain stimuli cause either cell cycle arrest or cell death, resulting in a reduction in colony number. Therefore, this methodology allows us to evaluate cell survival in a long term fashion. For the colony assay, 1 and 10 µM iRF were choosen, since these concentrations did not compromise melanoma cells viability after 48 h of treatment. Interestingly, 2 days of exposure to iRF was enough to cause an expressive decrease of B16F10 cell colonies (about 50%), even at low concentration of iRF (1 µM) - (Fig. 1E). It is important to mention that the size of colonies was smaller in the presence of iRF.

**Figure 2:**
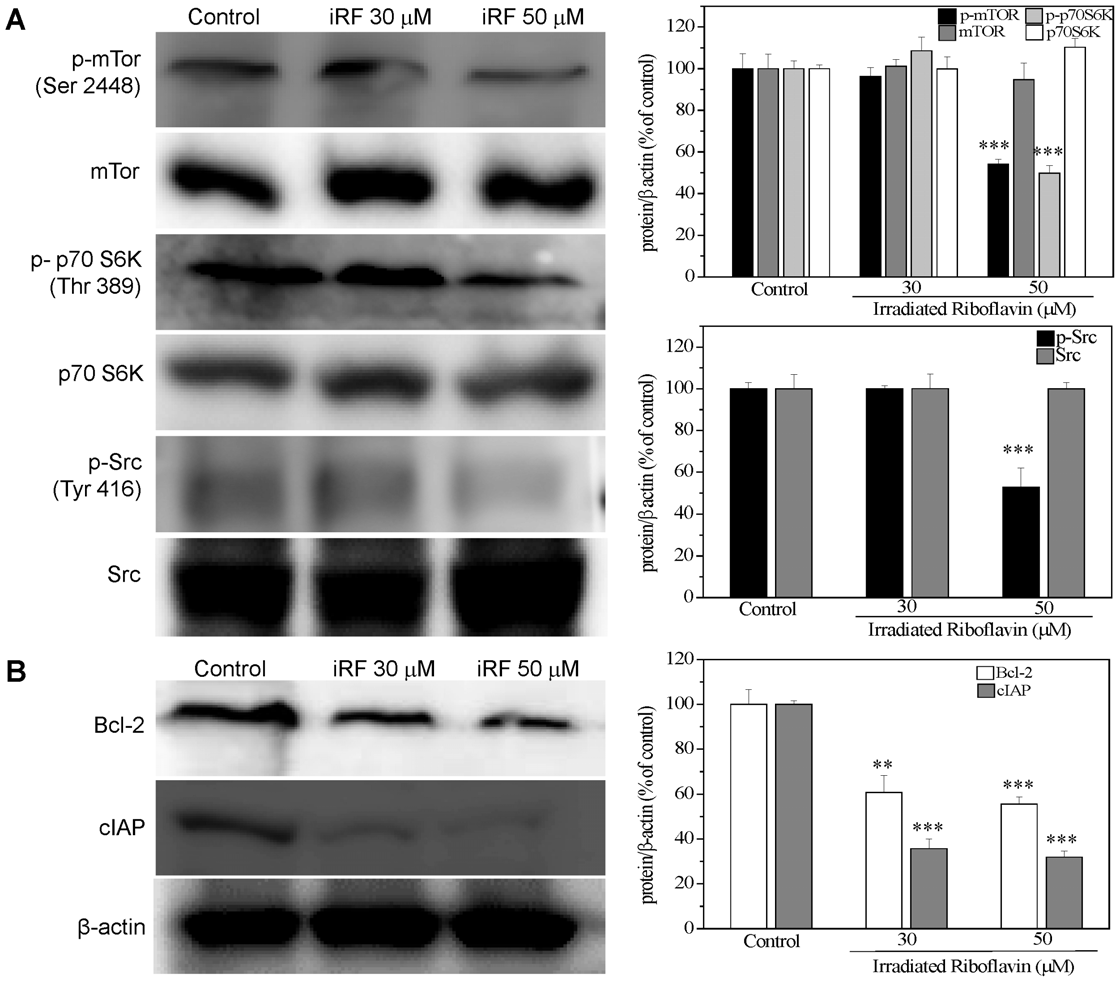
Effect of irradiated riboflavin on mTOR and Src activities and expression of antiapoptotic proteins Bcl-2 and cIAP. Analysis of the expression of p-mTOR (Ser 2448), mTOR, p70S6K and p-p70S6K (Thr 389) (A) and Bcl-2 and cIAP (B) in B16F10 treated with different concentrations of iRF for 24 h. The results are shown as mean ± SD of three independent experiments. p<0.001 versus control.

Due to the inhibitory action of iRF on melanoma proliferation, an important mediator for cell survival, mTOR, was analyzed in order to better understand the molecular mechanism by which iRF might act. iRF (50 µM) did not affect the expression of mTOR or p70 S6K but caused a decrease in phosphorylation of mTOR at the serine 2448 residue and its substrate p70 S6K (Thr 389), which in turn, results in inhibition of both proteins. (Fig. 2A). Importantly, inhibition of Src kinase was already detected in 30 µM iRF. Moreover, we examined the expression of two antiapoptotic proteins, Bcl-2 and cIAP. Figure 2B shows that iRF might induce apoptosis by downregulating the anti-apoptotic protein Bcl-2, which is one of central regulators of caspase activation, as well as, reducing the expression of cIAP, which specifically inhibits caspase-3.

### Melanoma Cell Migration is Reduced by iRF

**Figure 3:**
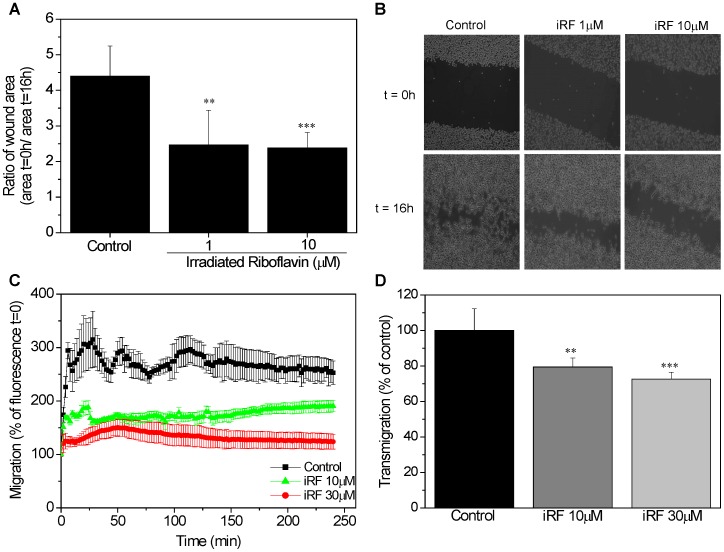
The effect of iRF on melanoma cell migration. (A) Scratch assay of B16F10 cells exposed to 1 and 10 µM of iRF. The images were acquired at 0 and 16 hours. The ratio of wound area at 16 h and at 0 h represent the inhibition of migration. (B) Representative pictures of scratch assay of each group after 0 and 16 h. (C) B16F10 cells were incubated with CellTracker green and then treated with different concentrations of iRF in serum free DMEM. Fluorescence was measured every 2 min for 4 h. (D) Migration capacity of B16F10 cells through the 2H11 endothelial cells monolayer was analyzed by transmigration assay using Fluoroblock™ inserts at 3 and 18 h. The results represent the means ± SD (n = 9). p<0.05, p<0.01, p<0.001 versus control.

One parameter of the aggressiveness of melanoma is its capacity for migration. To assess the effect of irradiated riboflavin on melanoma migration, we used an scratch assay, an *in vitro* wound healing assay. To enable “wound healing”, sub-toxic doses of iRF were used 1 and 10 µM. The scratch assay (Fig. 3A) revealed that iRF inhibits B16F10 cells migration. Furthermore, the concentration of 10 µM iRF was enough to reduce the wound area, resulting in a 0 h/16 h wound area ratio two times higher than the control (Fig. 3B).

To examine the effects of iRF on melanoma invasion, a Fluoroblok chamber assay was performed using 10 and 30 µM iRF. These concentrations affected the migration and transmigration of B16F10 cells without compromising melanoma viability as assessed by MTT assay. As showed in the figure 3C iRF treatment inhibited the cell migration.

Although transwell experiments are routinely used to study tumor cell migration, co-culture of melanoma cells with endothelial monolayers can provide better information about migration and extravasation efficiency of melanoma cells. Therefore, we grew monolayers of 2H11 endothelial cells on transwell filters to analyze if iRF can affect B16F10 migration through a monolayer of 2H11 endothelial cells. As shown in the figure 3D, iRF significantly reduced the migration of melanoma cells through the 2H11 monolayer.

### iRF Affects MMPs and TIMP-1 Activities and Expression

**Figure 4:**
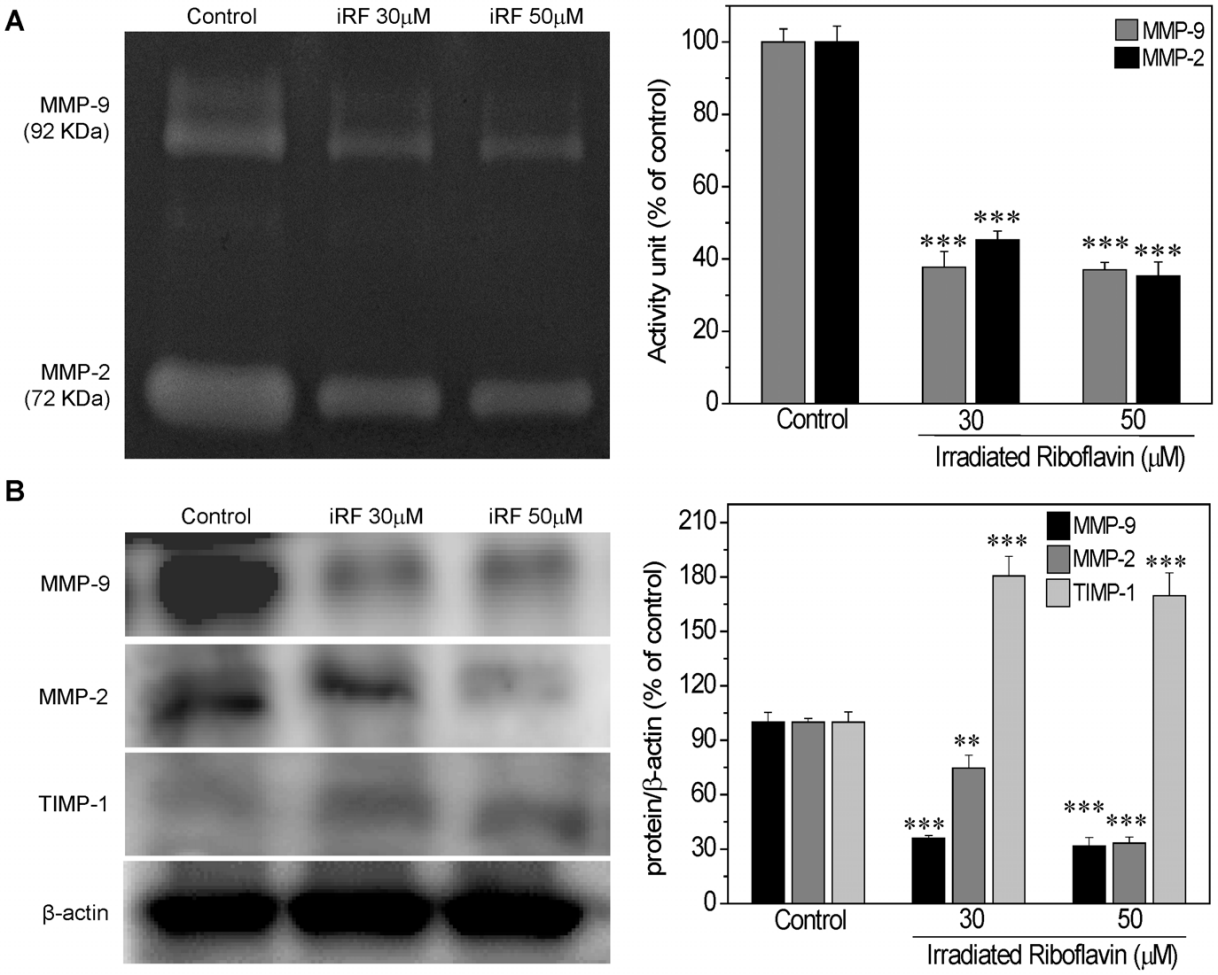
The effect of irradiated riboflavin on activity and expression of MMPs e TIMP-1. (A) B16F10 cells were treated with different concentrations of iRF for 24 h. Equal amounts of protein from each sample were applied to gel zymography. The activity of MMP-9 and MMP-2 was measured by densitometry of the gelatin zymography. (B) Western blotting analysis of the expression of MMP-9, MMP-2 and TIMP-1 in B16F10 treated with different concentrations of iRF for 24 h. The results are shown as mean ± SD of three independent experiments. p<0.01, p<0.001 versus control.

The migration capacity of melanoma cells is believed to be directly proportional to the MMP activity and inversely proportional to the TIMP activity. Thus, the activity and expression of MMP-9, MMP-2 and TIMP-1 were further investigated in B16F10 cells treated with iRF. In this experiment B16F10 cells were treated with 30 and 50 µM iRF, the same concentrations used in western blotting analysis, corresponding to viability reduction of 30% and 50%, respectively. Gelatin zymography (Fig. 4A) indicates that there is an inhibitory effect of iRF on the MMP-9 and MMP-2 activities. In addition, the expression of MMP-9 e MMP-2 was decreased and TIMP-1 was increased by RF treatment (Fig. 4B).

### Inhibition of Hedgehog Pathway Potentiates the Effect iRF in Melanoma Cells

**Figure 5:**
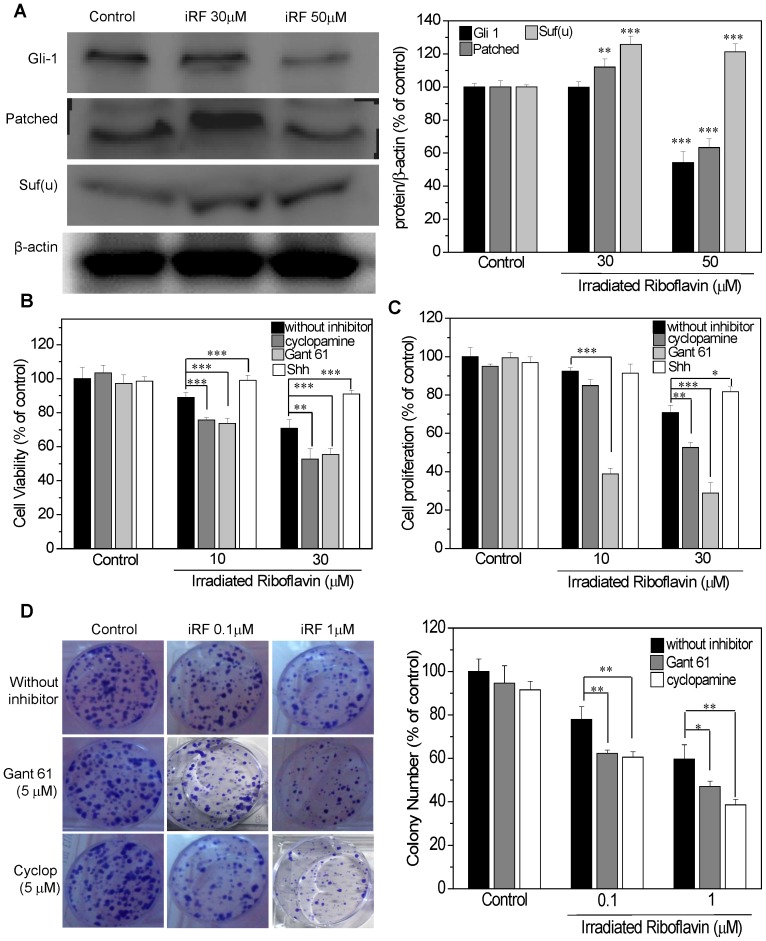
The effect of iRF on Hedgehog pathway. (A) Western blotting analysis of the expression of GLI1, PTCH and SUFU in B16F10 treated with different concentrations of iRF for 24 h. (B) Cell viability (MTT assay) of B16F10 cells with or without pretreatment with 5 µM cyclopamine, 5 µM Gant61 and 0.5 µg/mL SHH for 6 h and following iRF treatment for 24 h. (C) Cell proliferation BrdU assay with or without HH modulator pretreatment followed by iRF treatment and (D) representative pictures of colony assay and the number of colonies of B16F10 cells treated for 10 days. The results are expressed as the mean ± SD and are representative of three independent experiments to western blotting and n = 9 for others experiments. p<0.05, p<0.01, p<0.001 versus control.

To investigate whether iRF modulates HH pathway in B16HF10 cells, we analyzed the expression of important mediators of this cascade: GLI1, PTCH-1 and SUFU. Figure 5A shows that only 50 µM iRF treatment resulted in a negative modulation of HH pathway, as observed by decreased expression of GLI1 and PTCH-1, and upregulation of SUFU. To provide more evidence for the possible contribution of HH modulation by iRF, we examined the effects of HH pathway inhibitors (cyclopamine and Gant61) as well as the effect of an agonist (SHH) on melanoma cells viability and proliferation. In this experiment 10 and 20 µM of iRF were used, since the aim of this experiment was to evaluate a possible synergistic effect of combining iRF with HH pathway modulators. As shown in Figure 1D the cell viability in the presence of 20 µM iRF remained about 70%. Figure 5B and C show the viability and proliferation of melanoma cells, pretreated with either HH pathway inhibitors or the agonist, followed by iRF treatment. Inhibitors of HH cascade mildly improved the effect of iRF. On the other hand, when the cells were pretreated with the HH pathway agonist, the toxic effect of irradiated vitamin B_2_ was abrogated. In addition, we also evaluated the influence of HH inhibitors on the iRF effects in a colony formation assay. As this assay evaluates survival and the ability of clones to generate colonies in response to long term treatment, lower concentrations of iRF (0.1 and 1 µM) were used in combination with HH inhibitors. It is important to note that when the inhibitors were used alone, no large effect was observed on colony formation (less than a 10% decrease). In line with previous results, the use of HH inhibitors resulted in only a minor modulation of the iRF effects on B16F10 cell colony formation (Figure 5D). Taken together these results suggest that iRF effects on melanoma are due, at least in part, to impairment of HH pathway function. Accordingly, inhibitors of this pathway only mildly interfered with B16F10 cell proliferation and survival indicating that perhaps known HH inhibitors and iRF are targeting the same pathway.

### Flavins Inhibit *in vivo* Melanoma Metastasis

**Figure 6:**
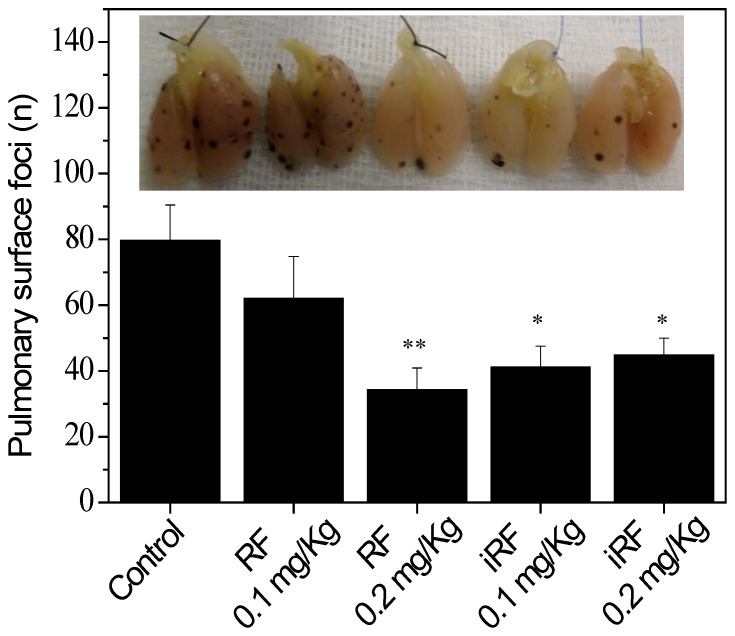
Effect of flavins on the number of B16F10 pulmonary tumor foci. After administration of 3×10^5^ B16F10 melanoma cells into the lateral tail vein,C57Bl/6 mice were treated intravenously with riboflavin or irradiated riboflavin 0.1 and 0.2 mg/kg, 6 times within 2 weeks. Mice were sacrificed 14 days after cancer cell injection and the number of tumor foci at the surface of the lungs was determined. The results are expressed as the mean ± SD (n = 8); * p<0.05, ** p<0.01 versus control.

To assess the effect of flavins on experimental metastasis, mice were treated with RF or iRF and metastasis in the lung were examined 14 days later. As shown in Figure 6, the number of pulmonary tumor foci was significantly reduced by flavin treatment as compared to the saline treated control group. Importantly, non irradiated riboflavin (0.2 mg/kg) displayed similar effect than iRF.

## Discussion

Besides being a precursor of redox coenzymes, riboflavin (RF) acts as an efficient biological photosensitizer upon activation with visible/UV light. Furthermore, RF and UV/Vis light exposed RF have been used in diverse settings such as pathogen reduction and inactivation in white blood cells in blood products [47], inactivation of botulinum neurotoxin [48], corneal collagen crosslink treatment of progressive keratoconus [49], [50], induction of cancer cell death [34], [37] and osteoblast differentiation [51].

In the present study we observed that iRF, which contains riboflavin, lumichrome 7,8-dimethylisoalloxazine, formylmethylflavin and carboxymethylflavin, was more cytotoxic to melanoma cells than to normal human keratinocytes. Our research group has reported a similar behavior of prostate cancer, normal human hepatocytes and rat prostate smooth muscle cells in response to iRF [34]. These results highlight that iRF seems to display selectivity for cancer cells.

**Figure 7:**
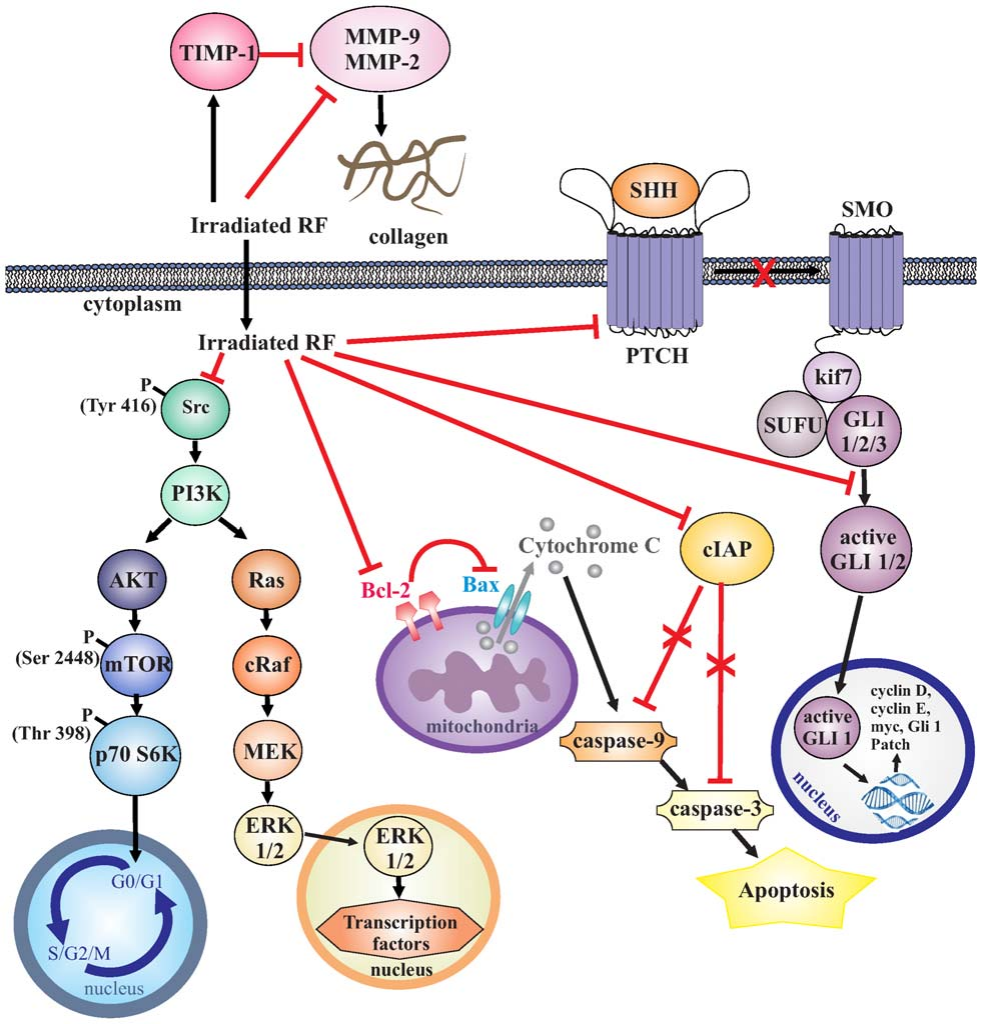
Schematic representation of the molecular mechanism by which iRF decreases the aggressiveness of B16F10 cells. iRF decreases the capacity of cell migration through inhibition of MMP-9 and MMP-2 activities as well as expression. Furthermore, the expression of a physiological inhibitor of MMPs, TIMP-1, was augmented by iRF. In addition, cell survival mediator, mTOR, was less active as well as Src kinase, an important key player of cell invasion. As we have shown previously in other cancer cell lines, iRF also induced B16F10 cells death by apoptosis since the expression of anti-apoptotic protein Bcl-2 is reduced. Bcl-2 acts by inhibiting Bax, a pro-apoptotic protein responsible to cytochrome-C release, and activation of caspases, leading cells to apoptosis. Furthermore, the expression of cIAP, a caspase 3, was diminished. HH is another pathway that seems to be important for iRF antimelanoma action. iRF inhibits HH pathway by downregulating GLI-1 and PATCH expression and overexpression of SUFU. GLI-1 can be sequestered by SUFU forming a complex with kif7 (Cos homologue in mice), thus keeping GLI-1 in the cytoplasm and preventing translocation to the nucleus, making it unable to activate the transcriptional program of the HH pathway.

In addition, we observed that iRF was able to negatively modulate the aggressiveness of melanoma cells, since proliferation, colony forming capacity, migration and invasion were strongly reduced after treatment. Based on these data, we examined different signaling pathways which could be related to the effect of iRF; these pathways are summarized in the Figure 7:

- mTOR pathway - iRF resulted in inhibition of both mTOR and its downstream molecule, p70 S6K. PI3K/AKT signaling is responsible for the phosphorylation of mTOR at Ser 2448. mTOR in turn phosphorylates p70 S6K at Thr 389. The focus on p70 S6K is based on its requirement for cell growth and G1 cell cycle progression [52], [53]. Li and colleagues [54], by using *in vivo* and *in vitro* studies, reported that 2-arylthiazolidine-4-carboxylic acid amides has an anti-cancer efficacy by inhibiting the PI3K/AKT/mTOR pathway;
- Src kinase inhibition - iRF decreased the phosphorylation of tyrosine 416, an activator residue of Src kinase. This nonreceptor protein tyrosine kinase is considered to be a key player in tumor progression providing oncogenic signals for cell survival, epithelial-mesenchymal transition, mitogenesis, invasion, angiogenesis, and metastasis. Aberrant expression and activation of Src occurs in breast, prostate, lung, and colorectal carcinomas, and are associated with poor clinical outcome. This stimulated interest in using SRC kinase inhibitors as therapeutic cancer agents, some of which have entered clinical experimentation [20].
- cIAP and bcl2 modulation - iRF caused downregulation of Bcl2 and cIAP. Bcl-2, an anti-apoptotic factor, levels are important for arbitrating cellular life-or-death balance [55]. Therefore, the reduced expression of Bcl-2 and cIAP (an inhibitor of caspase 3) after treatment of melanoma cells with iRF is indicative of apoptosis induction. Souza *et al*. [37] reported that iRF induces apoptosis of leukemia cells and De Souza Queiroz and colleagues [34] described similar effects in human prostate cancer cells (PC3).
- MMPs and TIMP-1 modulation - iRF disrupted melanoma cell migration by inhibiting MMPs and enhancing TIMP-1 expression. Profound changes in cellular cytoskeleton occur during cell adhesion, spreading and migration. These processes are often activated in cancer cells and promote invasion of neighboring tissues and spreading of metastatic lesions. To study the effects of new antitumoral agents, many biomarkers have been investigated including matrix metalloproteinases (MMPs) which are regulated by their tissue inhibitors (TIMP). Chen and colleagues [56] analyzed samples from patients and reported that there is a differential expression of MMP-9, which is present in early stage of benign lesions and a significant increase occurs in advanced malignant melanoma stages. This variation in MMP-9 activity might contribute to degradation of extracellular matrix, thereby promoting carcinogenesis and metastasis. MMP-2 and MMP-9 are considered good targets for anticancer drugs because they degrade gelatins, which are major components of basement membrane [34]. The data present here are in agreement with the notion that the reduction of extracellular matrix degradation leads to the inhibition of migration. This is additional evidence for iRF’s great potential as anti-cancer drug.
- Inhibition of HH pathway - HH has emerged as a signaling system that is a critical mediator of processes promoting tumor aggressiveness, such as metastasis, angiogenesis and acquisition of resistance to chemotherapy. Moreover, dysfunctions of HH mediators has been implicated to types of cancer which account for up to 25% of all human cancer deaths [57], [58]. Smoothened (SMO) inhibitor, cyclopamine decreases the ability of melanoma cells to form lung metastasis [40]. Effects of iRF on melanoma cells (B16F10 cells) are, at least in part, correlated with inhibition of HH signaling, as evident by the decreased the expression of key players in HH pathway signaling such as GLI1 and PTCH-1 and the increase of SUFU expression. However, since we did not observe an expressive toxicity of HH inhibitors when these were used in absence of iRF, this is indicative that B16F10 cells are quite resistant to these inhibitors.

Besides being important for growth and differentiation, HH is a mediator of different forms of oncogenesis. Stecca and collaborators [29] showed that systemic or local interference with HH signaling inhibits melanoma growth and prevents recurrence and metastasis. In the epidermis, HH pathway activation promotes proliferation and renders cells resistant to stimuli promoting their cell cycle exit and terminal differentiation [59], [60]. In mice, overexpression of SHH, activates SMO, GLI1 or GLI2 and promotes development of basal cell carcinoma like tumors [61]. The canonical HH pathway is initiated by HH ligand binding to the transmembrane receptor PTCH that in turn releases its inhibition of SMO [62]. Activated SMO causes the dissociation of SUFU from GLI, which translocates to the nucleus and activates the transcriptional program of the HH pathway [63]. SUFU inhibits GLI-mediated transcriptional activation in the nucleus by recruiting a histone deacetylation complex to GLI target genes [64], [65]. Thus, the SUFU overexpression after treatment of the cells with iRF could interfere with GLI1 transcriptional activity. To reinforce our hypothesis that the inhibition of HH is important for iRF action in melanoma cells, the effects combinating the inhibitors of this pathway with RF was examined. Cyclopamine and Gant61, inhibitors of SMO and GLI1, respectively, were used. The combination of these inhibitors with iRF led to greater decrease in viability and proliferation of melanoma cells. Therefore, we can hypothesize that iRF treatment sensitizes B16F10 cells for HH inhibitors. One possibility is that iRF could affect some lipid domains (lipid rafts) in plasma membrane of B16F10 cells that result in alteration of HH signaling. Previously, we proposed that part of the anti-leukemia action mechanism of iRF is due to lipid rafts rearrangement (de Souza et al., 2006). However, to confirm this hypothesis, additional experiments need to be done.

Given, the very interesting findings on the molecular action of flavins on melanoma cells, we were prompted to examine the action of these compounds *in vivo*. Differently from observed *in vitro*, RF as well as iRF displayed a strong inhibitory effect on lung metastasis formation. This apparent discrepancy between *in vitro* and *in vivo*, was not due to toxic effects of RF in melanoma cells, but might be due to the metabolization of RF *in vivo*, which also provides photoproducts as main catabolites. It has been shown by Ohkawa *et al* (1983) [35] and Chastain and McCormick (1987) [36] that RF photodecomposition can occur in the human body.

Taken together, the data presented here, provides evidence of the potential of flavins to diminish the aggressiveness of melanoma, and therefore, it might be interesting to apply these photoproducts in pharmaceutical formulations for treating melanoma. Moreover, to our knowledge this is the first report of the antimetastatic properties of flavins *in vivo*.
